# Relating Demographic Characteristics of a Small Mammal to Remotely Sensed Forest-Stand Condition

**DOI:** 10.1371/journal.pone.0091731

**Published:** 2014-03-12

**Authors:** Hania Lada, James R. Thomson, Shaun C. Cunningham, Ralph Mac Nally

**Affiliations:** School of Biological Sciences, Monash University, Melbourne, Victoria, Australia; Institute of Agronomy, University of Lisbon, Portugal

## Abstract

Many ecological systems around the world are changing rapidly in response to direct (land-use change) and indirect (climate change) human actions. We need tools to assess dynamically, and over appropriate management scales, condition of ecosystems and their responses to potential mitigation of pressures. Using a validated model, we determined whether stand condition of floodplain forests is related to densities of a small mammal (a carnivorous marsupial, *Antechinus flavipes*) in 60 000 ha of extant river red gum (*Eucalyptus camaldulensis*) forests in south-eastern Australia in 2004, 2005 and 2011. Stand condition was assessed remotely using models built from ground assessments of stand condition and satellite-derived reflectance. Other covariates, such as volumes of fallen timber, distances to floods, rainfall and life stages were included in the model. Trapping of animals was conducted at 272 plots (0.25 ha) across the region. Densities of second-year females (i.e. females that had survived to a second breeding year) and of second-year females with suckled teats (i.e. inferred to have been successful mothers) were higher in stands with the highest condition. There was no evidence of a relationship with stand condition for males or all females. These outcomes show that remotely-sensed estimates of stand condition (here floodplain forests) are relatable to some demographic characteristics of a small mammal species, and may provide useful information about the capacity of ecosystems to support animal populations. Over-regulation of large, lowland rivers has led to declines in many facets of floodplain function. If management of water resources continues as it has in recent decades, then our results suggest that there will be further deterioration in stand condition and a decreased capacity for female yellow-footed antechinuses to breed multiple times.

## Introduction

Fast, extensive change now is the dominant characteristic of ecological systems across the world. This is due to climate change, many direct actions of humans, and myriad indirect effects arising from those actions [1], [2], [3]. Rates of change are so rapid and extents so large (e.g. forest dieback increased from 45% to 70% in 16 years over 100 000 ha of Murray River floodplains in Australia [4]) that new methods for evaluating and tracking ecosystem change are required to anticipate and potentially to mitigate undesirable ecological outcomes. Traditional methods of field-based surveys require many years to cover large areas but the time-scales of threats (land clearance, fires, dam building) usually are much shorter than the intervals between assessments [5], [6].

Remote sensing offers the capacity to represent dynamically, and at appropriate spatial scales, the condition of surrogates (e.g. land use or vegetation condition) and then to project changes in biodiversity as the surrogate itself responds to anthropogenic pressures (e.g. land-use change) or natural processes (e.g. regional climate change, forest senescence). For example, in the Cumberland Mountains in the USA, 200 000 ha were assessed remotely as a potential habitat for cerulean warblers *Setophaga cerulean* [7]. The constructed model then was employed to evaluate the effects of proposed coal surface mining on warbler habitat [7]. At the tens of ha scale, aerial photography and surveys of birds and macroinvertebrates were used to monitor decline of seagrass coverage and population crash of seagrass-dependent species in a South African marine reserve [8].

Remotely sensed data have been used to estimate forest stand condition (an indicator of dieback) over >200 000 ha in the southern Murray-Darling Basin in south-eastern Australia [9]. In floodplain forests, changes in stand condition can be assessed in response to water management [4]. Mac Nally et al [10] found that abundance, effective species richness and breeding of birds were related to modeled stand condition; they predicted negative consequences of climate change on stand condition and avifauna on these floodplains.

The yellow-footed antechinus *Antechinus flavipes* Waterhouse is the only native, terrestrial, carnivorous mammal (a marsupial) on these floodplains. This species is most abundant in floodplain forests [11] but has been lost from floodplains of the lower, more arid sections of the Murray River [12]. Understanding of the reasons for changes in antechinus population characteristics (densities, survival) is important for planning management actions. Capture rates of antechinus are related to the occurrence of, and proximity to, floods [13]. Adult male antechinuses die after synchronized breeding in winter (before young are born), and many females die too (after weaning), with only a fraction of females surviving to produce offspring in their second year [14]. The occurrence of second-year females with suckled teats means that they survived to breed again and probably weaned offspring. Lada *et al.* [15] considered capture rates of females and of second-year females with suckled teats as excellent measures of realized habitat quality and thus the probability of population persistence in a given location.

If there are relationships between modeled stand condition and population characteristics of antechinus (e.g. abundance of females), these could provide a tool for rapid, remote assessment of antechinus across vast landscapes.

In this paper we explored the relative importance of modeled stand condition and of in-stand variables in explaining population characteristics of antechinus.

## Materials and Methods

### Study areas

**Figure 1:**
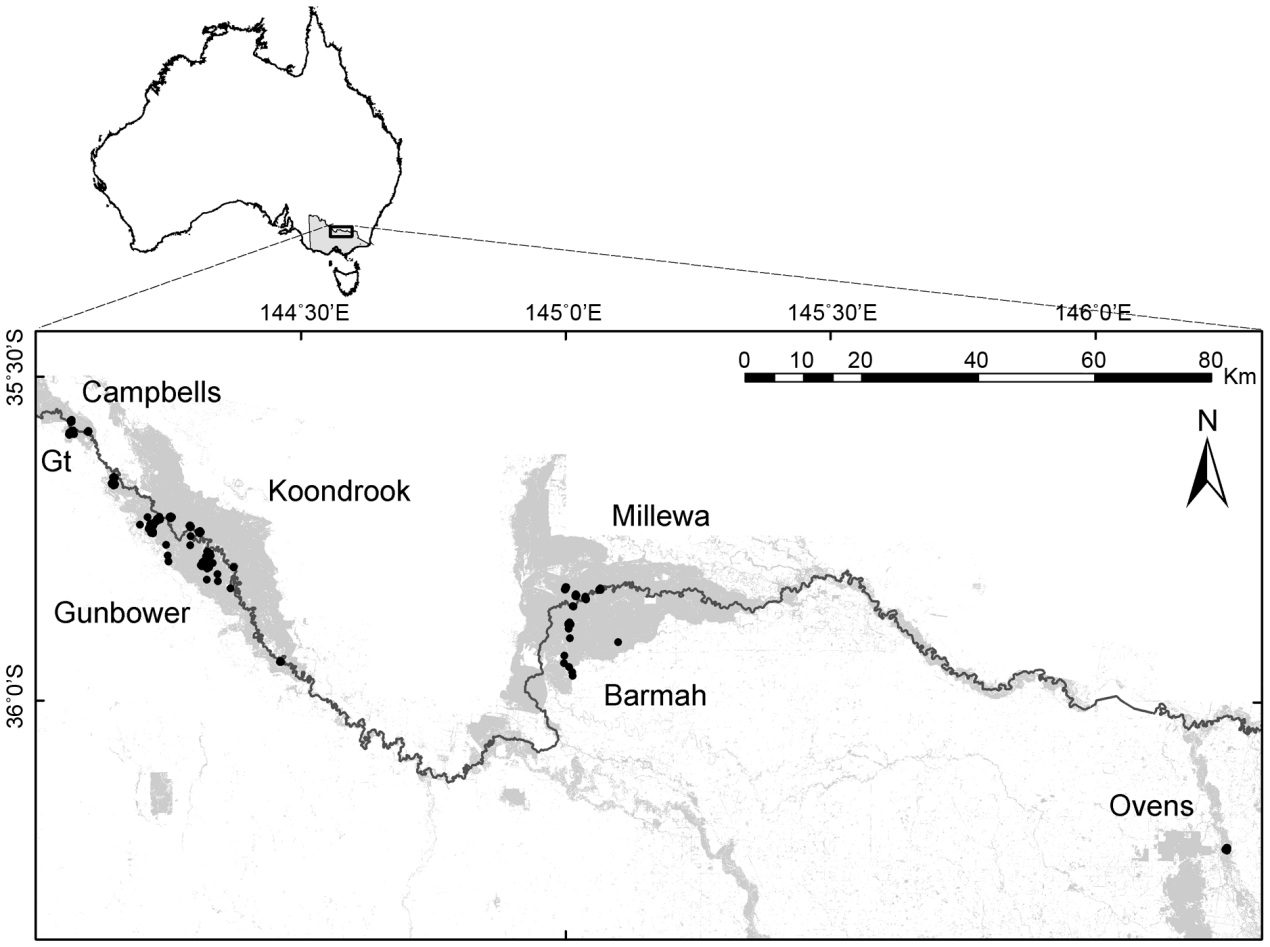
Location of study sites on the middle Murray River floodplain, south-eastern Australia. Grey areas represent extant forests. Black dots represent locations of 1–27 study sites. Extensive forest areas are labeled, with Gt indicating Guttrum forest. Gunbower and Barmah were surveyed in 2004, 2005 and 2011; Campbells, Guttrum, Koondrook, Millewa and Ovens – in 2004 and 2005.

The study was conducted in seven floodplain forests and woodlands of river red gum (*Eucalyptus camaldulensis* Dehnh.) in south-eastern Australia (Fig. 1 and [11]). We investigated floodplains of the unregulated Ovens River and floodplains of the highly regulated Murray River, including Barmah, Millewa, Koondrook, Gunbower Island, Guttrum and Campbells Island Forests (Table S1 in File S1).

*Eucalyptus camaldulensis* is a mono-dominant tree species on these floodplains, forming open forests and woodlands (trees 10-30 m tall, 20–45% projective foliage cover) [16]. Groundcover is fallen timber, litter, low shrubs, sedges and grasses [17]. Mean annual rainfall over the study area ranges spatially from 395 (± 115, temporal SD, based on 30 years) to 624 (± 186, based on 23 years) mm yr^−1^ and mean monthly maximum temperatures range from 12.9°C for the coldest month to 31.8°C for the hottest month during the year [18]. Barmah, Millewa, Koondrook and Gunbower Island Forests are Ramsar-listed wetlands. The Murray River wetlands experienced regular floods prior to river regulation, with extensive floods 45 years per century [19]. Between 1980 and 2011, the frequency of extensive floods was halved compared with pre-regulation frequency [20] due to water management (dams, locks, irrigation channels). The floodplains have experienced grazing, logging and removal of fallen timber for > 130 years [21], along with significant water extractions [22]. Since 2000, small management floods have been used in selected areas of floodplains to improve ecosystem condition [13].

### Data sources for *Antechinus flavipes*

Antechinuses were captured in the austral summer, autumn and winter in 2004, 2005 [11] and 2011 at 272 randomly selected square 0.25 ha sites (Table S1 in File S1). Sites visited in 2004 were revisited in 2005 [11] and had been randomly selected in 24 within-forest areas (500 × 500 m) prior to the availability of the model of stand condition (see below). Sites for 2011 surveys were randomly selected with Hawth's Tools (http://spatialecology.com) using an existing map of stand condition [23]. We obtained sites from the full range of stand condition, avoiding spatial clustering of sites with similar stand condition. Sites with representative volumes of fallen timber were chosen because antechinus numbers increase with higher volumes [24]. Sites were > 300 m apart to avoid recaptures from other sites. Five or six small-mammal (Elliott) traps were placed along logs and beneath trees at each site for 1–5 nights. Traps were set for at least two nights on 83% of visits to sites. Differences in trapping effort arose because of early collection of traps on sites where individuals were repeatedly recaptured or access was impossible because of logging and management releases of water; differences were accounted for statistically (see below, this section). This trapping effort was sufficient to capture most, if not all, antechinuses present on site because recapture rates were high and animals were seen moving so extensively within sites that they were unlikely to miss locations of traps [11]. We did not trap in spring to avoid possibly stressing lactating females [25]. We used capture rates ( =  numbers of different individuals captured as a function of trapping effort, hence a binomial response variable) as a measure of density of antechinuses. We analyzed capture rates for all distinct individuals, males, females, second-year females, second-year females with suckled teats.

### Ethics statement

This work was carried out under permits BSCI/2011/03, BSCI/2003/02 (Monash University Biological Sciences Animal Ethics Committee, permits 10005842 and 10002325 (Department of Sustainability and Environment). Traps contained lure and bedding, were protected by plastic bags and covered with bark. Animals were released at the point of capture.

### Stand-condition scores

Stand-condition scores (see Table 1) were obtained from GIS rasters of modeled stand condition of river red gum forests in 2006 (for 2004 and 2005 trapping) and 2010 (for 2011 trapping) [23], [26]. Stand condition was estimated from the three variables: percentage live basal area, plant area index (PAI) and crown extent, which are known to be reliable indicators of condition in *E. camaldulensis* stands (see Methods S in File S1and [27] for further details). Stand condition was calculated at a resolution of 1 ha because this is similar to the home range for yellow-footed antechinus (this home range is inferred from trapping data in our study area in 1999–2005, and is consistent with radio-tracking studies elsewhere by [28]). The stand condition model for 2006 was built from extensive on-ground surveys (*N*  =  140 stands) in that year and had high predictive power in an independent, follow-up validation survey in 2007 (*N*  =  42 stands, *R* ^2^  =  0.78). The stand condition model for 2010 was built from on-ground surveys in that year (*N*  =  175 stands).

**Table 1:** Predictor variables used in the analysis of capture rates of the yellow-footed antechinus *Antechinus flavipes* in river red gum woodlands in 2004, 2005 and 2011 in south-eastern Australia.

	Year 2004	Year 2005	Year 2011	
Predictor variable	Mean ± SD (in *n* forests)	Mean ± SD (in *n* forests)	Mean ± SD (in *n* forests)	Data source
Stand-condition score	7.23 ± 0.72 (7)	7.22 ± 0.74 (7)	6.93 ± 0.97 (2)	GIS rasters of modeled stand condition; 2006 model [23] for 2004 and 2005 trapping; 2010 model [26] for 2011 trapping
Volume of fallen timber (m^3^/ha)	65.41 ± 36.11 (7)	66.69 ± 36.01 (7)	44.74 ± 29.82 (2)	All logs with diameters ≥ 10 cm on 0.25 ha sites [11]
Distance to floodwaters (km)	3.16 ± 4.14 (7)	3.03 ± 4.11 (7)	0 ± 0 (2)	Maps of inundations and field observations in 2003–2011
Number of orb-weaving spider webs	NA	NA	4 ± 5.1 (2)	Webs counted within 2 m of each trap line
Annual rainfall in previous year (mm)	535.2 ± 114.5	407.7 ± 73.4	657 ± 20.4	Data from three weather stations in 2004 and 2005, two stations in 2011 [18]
Juvenile dispersal				Categorical variable; whether trapping was in January (juvenile dispersal) or in June and July (breeding season)
Post-juvenile dispersal				Categorical variable; whether trapping was in March to May (post-juvenile dispersal) or in June and July (breeding season)

NA  =  not collected.

### Other environmental variables

Volume of fallen timber (Table 1) was estimated at each 0.25 ha site by considering all logs with a diameter ≥ 10 cm [11]. Distance to floodwaters (for floods occurring anytime between previous breeding season and current trapping) was measured from maps of inundations informed by field observations. The number of large webs of golden orb-weaving spiders *Nephila* sp. were counted within 2 m of each trap line. We considered the number of webs as a proxy for abundances of spiders and possibly of other macroinvertebrates, which are food for antechinus [12]. We tested the suggestion of Lada *et al.* [11] that these spiders may be associated with higher densities of antechinus. Values of annual rainfall of the previous year (weather conditions pre-, post- and during-breeding season of the previous generation and pre-weaning conditions for the current generation) were obtained from Bureau of Meteorology [18].

We included two indicator variables to determine whether capture rates were affected by a life stage: juvenile dispersal variable, which was used to compare capture rates in January (juvenile dispersal) to those in June and July (breeding season); and post-juvenile dispersal variable (capture rates in March to May vs during breeding season).

### Statistical analyses

We used logistic regression in WinBUGS v1.4 [29] to examine the relationship between capture rates of antechinus and environmental variables including stand condition. The model was:
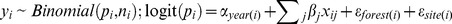



Here, *y_i_* is the total number of individuals (i.e. males and females) captured over *n_i_* trap-nights (i.e. each trap-night regarded as a Bernoulli trial, so that *n_i_*  =  the number of available traps) at site *i*, and *p_i_* is the corresponding capture rate to be estimated (i.e. probability of success for a single trap-night at a given site, in a given year). The αs are year-specific intercepts, logit-transformed mean capture rates for 2004, 2005 and 2011, and *β_j_* is the regression coefficient for the *j^t^* ^h^ predictor variable. The *ε*s are random effects for forest and site. Forest random effects were assigned exchangeable, normal prior distributions (Gelman 2005) with means 0 and variances, *σ_forest_* ^2^. The site random effects were modeled with a spatial disc function such that the correlation between sites declined nearly linearly with distances up to 500 m (typical home range for antechinus is 1 ha). We assigned uniform priors (0, 5) to the standard deviations of the random effects terms, *σ_site_*, and *σ_forest_* [30].

We used Bayesian model selection, implemented with the reversible jump MCMC add-on to WinBUGS [31], to identify a subset of the candidate predictor variables that should be included in the best model, or, equivalently, to identify which of the linear coefficients *β* are non-zero (see Methods S1 in File S1). The posterior probability of a non-zero coefficient Pr(*β_j_* ≠ 0) is a measure of the evidence that variable *j* is a predictor of the response. We considered Pr(*β_j_* ≠ 0) > 0.75, which is equivalent to a threefold increase in the prior odds (which were unity), to be evidence that a predictor has a strong effect on the response variable [32]. We used exchangeable prior distributions for the coefficients *β_j_* ∼ N(0, *σ_β_*); *σ_β_* ∼ Uniform(0, 2). We re-fitted models with a range of plausible upper limits on *σ_β_* (0.3, 1, 2, 4) and obtained similar results.

Models were estimated with three Markov chain Monte Carlo (MCMC) chains run for 100000 iterations following burns-in of 50000 iterations.

We checked the adequacy of the model structure by posterior predictive checks using the χ^2^-discrepancy statistic. We also calculated *pseudo-R^2^* values (proportion of deviance explained divided by maximum) as a measure of model fit. We performed 10-fold cross-validation on models to check that estimated relationships were not spurious and to evaluate likely predictive capacity. The data were split into 10 sets of sites (folds), and each fold, comprising all surveys at each site, served as test data for models built with the remaining sites. Sites were semi-randomly allocated to folds, but sites within 500 m of each other were allocated to the same fold so that predictions were not informed by spatial autocorrelation. We calculated cross-validation *pseudo-R^2^* [33] for the combined hold-out data, and used random permutation of predictions to calculate the probability of obtaining equal or higher *pseudo-R^2^* values by chance.

### Estimates of temporal trends in fractions of stand condition

To track changes in stand condition across these floodplains, a temporal series (1990, 2003, 2006, 2009 and 2010) of condition maps for the region was compiled from previous work (see Methods S1 in File S1).

## Results

There were 0–5 distinct individuals of *Antechinus flavipes* captured per site in 2004, and 0–7 individuals in 2005 and 2011. No more than two second-year females were caught on a given site. At least 6% of second-year females failed to produce any offspring (i.e. they did not have any suckled teats). There were 557 captures of antechinus (same-site recaptures discounted) with the average of 1.1 ± 1.4 SD individuals per site. There was strong evidence that capture rates of second-year females and of second-year females with suckled teats increased with increasing stand condition (Table 2). There was strong evidence that the capture rates of total number of individuals increased with the volume of fallen timber, and that capture rates of males were lower during the post-juvenile dispersal period than during the breeding period. There was no evidence that other environmental variables (distance to floods of current and previous year, rainfall previous year, golden orb-weaving spider webs) influenced capture rates (Table 2).

**Table 2:** Results of Bayesian regression analyses [posterior mean regression coefficient, *β*, and probability of non-zero coefficient, Pr(*β*≠ 0)] of capture rates of the yellow-footed antechinus *Antechinus flavipes* in river red gum woodlands in 2004, 2005 and 2011 in south-eastern Australia with respect to environmental variables and stage of life cycle.

	Total		Males		Females		F2		F2 with teats	
Variable	Mean ±SD	Pr	Mean±SD	Pr	Mean±SD	Pr	Mean±SD	Pr	Mean±SD	Pr
*α*_04_	−2.97±0.25		−3.43±0.25		−3.96 ±0.29		−5.99±0.76		−6.88±0.77	
*α*_05_	−2.31±0.24		−2.71±0.30		−3.47±0.29		−5.14±0.59		−5.59±0.66	
*α*_11_	−2.16±0.41		−2.56±0.51		−3.61±0.46		−5.52±1.01		−6.03±1.01	
Condition	−0.01±0.03	0.22	−0.12±0.11	0.69	0.07±0.10	0.54	**0.45±0.29**	**0.86 (0.85)**	**0.38±0.40**	**0.75 (0.77)**
FloodDist	−0.01±0.04	0.27	0.00±0.03	0.17	0.00±0.04	0.34	0.00±0.03	0.14	0.00±0.05	0.20
Logs (m^3^h^−1^)	**0.13±0.08**	**0.87 (0.82)**	0.05±0.07	0.49	0.08±0.09	0.65	0.01±0.04	0.16	0.00±0.03	0.17
RainPrevYr	−0.03±0.09	0.39	0.00±0.16	0.51	−0.04±0.09	0.42	−0.17±0.21	0.63	−0.02±0.09	0.26
Webs	0.00±0.02	0.19	0.00±0.01	0.10	0.00±0.02	0.26	0.00±0.01	0.09	0.00±0.01	0.09
JD	0.06±0.22	0.46	−0.12±0.28	0.46	0.13±0.35	0.56	0.07±0.33	0.34	0.26±0.68	0.51
PostJD	−0.14±0.18	0.61	**−0.46±0.25**	**0.93 (0.93)**	0.01±0.07	0.41	−0.39±0.57	0.53	0.00±0.08	0.27
Pseudo-*R* ^2^	0.56 (0.09)		0.42 (0.10)		0.43 (0.05)		0.28 (0.07)		0.33 (0.07)	

Pr(*β*≠ 0) values in parenthesis are averages of cross-validation fits [shown only for variables with Pr(*β*≠ 0) > 0.75]. Response variables: F2  =  second-year females, F2 with teats  =  second-year females with suckled teats. Covariates: Condition  =  modeled stand condition at 100 m resolution, Webs  =  number of webs of golden orb-weaving spiders, FallenTimber  =  volume of fallen timber, FloodDist  =  Euclidean distance to flood waters, RainPrev6mon  =  rainfall over 6 months preceding the month of trapping, RainPrevYr  =  annual rainfall previous year, JD  =  whether trapping occurred during juvenile dispersal phase, postJD  =  whether trapping occurred between juvenile dispersal and breeding stages. Pr  =  probability that the covariate is a predictor of the response. Mean  =  regression coefficient. SD  =  standard deviation of regression coefficient. Pseudo*-R^2^* is the proportion of the binomial deviance [–2log(likelihood)] explained by the fitted model divided by the maximum possible value, values in parentheses are the corresponding values for 10-fold cross validation.

Variable-selection results in cross-validation tests were consistent with values obtained for the full data (Table 2). For all variables that had Pr(*β_j_* ≠ 0) > 0.75 in the full model, Pr(*β_j_* ≠ 0) values exceeded 0.75 in at least 8 of 10 cross-validation iterations and were never < 0.6 (hence > 0.5, the prior probability). Predictive capacity of models was low (*pseudo-R^2^* ≤ 10%) but better than random for all response variables [Pr(observed or lower *pseudo-R^2^* | random) < 0.001].

**Figure 2:**
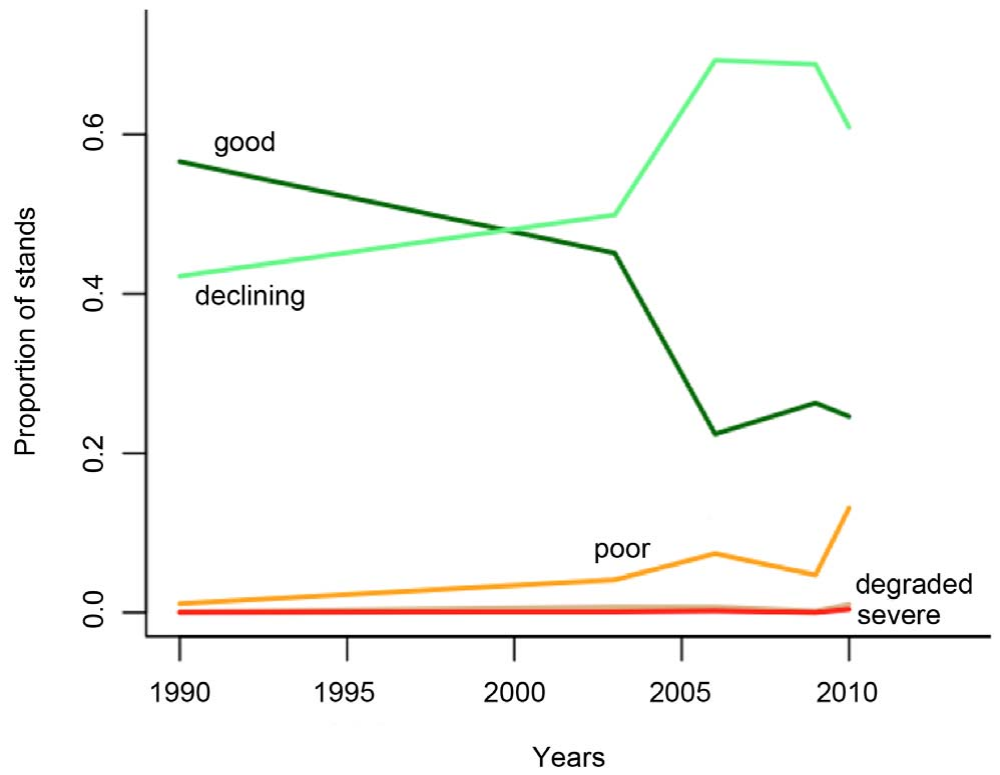
Proportion of floodplain forest in different stand-condition categories along the mid-Murray River between 1990 and 2010. Stand condition was predicted from maps that were built from ground surveys and Landsat imagery [4], [23], [26]. Key: (1) good, dark green; (2) declined, light green; (3) poor, orange; (4) degraded, brown; (5) severe, red. Redrawn from [10].

The best estimate of the percentage of the floodplain forest stands in ‘good’ condition (SCS > 8, see example in Fig. S1) declined from 57% in 1990 to 25% from 2006 onwards (Fig. 2, redrawn from [10]), with a very rapid decline between 2003 and 2006 following almost a decade of much-below-average rainfall. Much of the change was into the ‘declined’ class until 2010, when there was a substantial rise in the percentage of ‘poor’ condition forest (Fig. 2, and see example in Fig. S1).

## Discussion

The first and second generation of *Antechinus flavipes* differed in their responses to modeled stand condition (Table 2). Modeled stand condition was the best predictor for relative abundances of second-year females and second-year females with suckled teats, but not for abundances of males or for total females (first-year and second-year females combined). That the probabilities of capturing second-year females and second-year females with suckled teats were related positively to stand condition, while densities of males were unrelated, is consistent with the species' biology. After weaning in summer, juvenile males disperse from natal areas and, during the breeding season, roam widely searching for females with which to mate [28], [34]. Females are much more philopatric and remain near natal areas and with female relatives [35]. The response of second-year females and second-year females with suckled teats to modeled stand condition is of greater demographic importance than is the lack of male response, given that population dynamics effectively are driven by the survival of females and their offspring (i.e. total breeding failure in one year would extirpate a population if there were no subsequent immigration [15]). The lack of response in total females to stand condition suggests that stand condition is a better predictor of past breeding success (females with suckled teats) and survival of females to their second year (second-year females) than of future breeding potential (all females). This idea of differences in past breeding success and future breeding potential in relation to stand condition needs to be explored in other species of vertebrates.

The relationship between densities of second-year females and stand condition may reflect an association between stand condition and resource provision for antechinus, but more likely reflects the positive effects of flooding on both stand condition and floodplain productivity [4], [13]. Proximity to flooding is a strong predictor of second-year female densities [13] and females move into the regions closest to floods [35]. Emergence of large-bodied macroinvertebrates (beetles and spiders) after the flood recession almost certainly provides abundant large prey for antechinuses [36]. While stand condition may appear to be a proxy for flooding frequency, this simple relationship is complicated because the densities of males increase with proximity to flooding [13] but not with higher levels of stand condition (Table 2). To determine why stand condition is a strong predictor of second-year female densities, we need to establish the relationship between stand condition and the availability of food and shelter (on or in trees) for antechinuses. Stand condition might affect ground habitats through shading and the amount of fallen timber.

These floodplain forests have been subjected to extensive logging, grazing and water extractions, which led to changes in forest structure from one dominated by large, spreading trees with mixed-aged patches to one of relatively young, same-age stands with few large trees [20]. Tree recruitment is now rare because of reduced flood frequency, grazing and salinity of soil and groundwater [20]. Sometimes water is returned to floodplain forests to mitigate extensive tree dieback (70% dieback in 2006 [4]) or for safety of towns and upstream dams. Some management floods are detrimental to both trees and to antechinus. Flows during summer support irrigated agriculture but were rare before widespread river regulation [37]. Management floods may be maintained for up to four months, much longer than historical floods [38], which almost certainly limits foraging opportunities for antechinus females carrying young and delays macroinvertebrate emergence. Recruitment of river red gum is highest following winter to early spring floods [39] because these allow development of deep root systems to avoid subsequent water deficits. Given the common need for spring floods of antechinus and of river red gum, stand condition may provide a more integrated assessment of flooding history, and thus productivity, for antechinus on floodplains.

Maps of stand condition over 100 000 ha have been produced annually from on-ground surveys and remotely-sensed data since 2009 [4]. These maps may be used to prioritize and monitor releases of limited amounts of management water onto floodplains at temporal and spatial scales to maximize the positive effects on stand condition, which may also lead to positive effects on survival of antechinus mothers.

There is a lack of information on how changes in land-use and management actions, such as management floods, affect (meta) population dynamics [40]. Typically, at least one of the many parameters in metapopulation viability analyses is poorly known. For antechinus, we have knowledge on effective dispersal from genetic analyses [41]. Unfortunately, capture rates of antechinus (especially second-year females, 0.002 in 2004, 0.006 in 2005, Table S2 in File S1) are too low to accurately estimate the densities of second-year females in patches of habitat of different (modeled) condition. One or two captures can cause large deviation from expected capture rates. For example, simulated captures for second-year females based on actual trapping efforts and the predicted capture probabilities yielded pseudo*-R^2^* values lower than the observed 7% (values for 10-fold cross validation in Table 2) with probability 0.49. That is, even if predicted capture rates matched the true underlying probabilities of capture perfectly, we would not expect higher pseudo*-R^2^* values. However, our results provided strong evidence that densities of second-year females generally will increase with increasing stand condition, all else being equal. Of course, there may be other limiting factors, such as macroinvertebrate abundance (food availability for antechinus) and fallen timber volumes (which were not correlated with stand condition) that will cause local variation in antechinus densities independently of stand condition.

Our findings have relevance to management of floodplains in semi-arid areas. River regulation and water diversions are associated with similar tree population declines on floodplains in southwest of the USA [42] and in Spain [43]. In Australia, the widespread decline in river red gum condition across the Murray-Darling Basin has been addressed poorly by very limited allocations of overbank flows across all of the floodplains [44]. The south-eastern Australia was gripped by severe drought (1997–2010) and subject to consistently increasing temperatures [45]. Severe droughts are likely to recur under predicted climate change [46]. During the severe drought, densities of antechinus were higher closer to floods [13], and stands in good condition were restricted to areas closest to the river channel, to permanent wetlands or where management floods had occurred [4]. These results, combined with our results of a positive relationship between stand condition and inferred breeding success of antechinus (females with suckled teats) suggest advantages for increased flood allocations. Depriving floodplains of water most likely will lead to further decreases in stand condition and to decreased ability of antechinus mothers to breed again. Floodplains may offer sanctuaries for antechinus populations during droughts. This is in contrast to box-ironbark forests, where the antechinus population in Chiltern [highest capture rate of any forests in 2004 and 2005; 11] crashed to small numbers in 2011, most likely because of low rainfall in the preceding years [25].

Our results suggest that remote sensing offers a means to assess rapidly habitat quality for one mammal species over large areas. We linked a stand condition model, which originally was developed to assess health of river red gum forests, to population dynamics of antechinus and birds [10]. Remotely-sensed estimates of stand condition and structure have been related to biodiversity measures in many parts of the world (see review by [47]), and the capacity to make larger-scale estimates of biodiversity status is an important advance. Our results, when coupled with strong positive responses of birds to variation in stand condition in the same floodplain forests [10], contribute to this growing body of knowledge that allows upscaling from plot-scale measurements to landscape and regional scale estimates of ecosystem condition and biodiversity status. We present an approach that is potentially transferable to other species and ecosystems, and that may be a means to dynamically link both anthropogenic and natural environmental change to habitats of species and measures of species' status and trends.

## Supporting Information

Figure S1 Examples of stand condition states: good, poor and severe.
(TIFF)

File S1 Contains the following files: **Methods S1.** Model selection with reversible jump MCMC. Stand-condition scores. Estimates of temporal trends in fractions of stand condition. **References S1. Table S1** Study area and site information. **Table S2** Mean trapping rates of the yellow-footed antechinus in 2004, 2005 and 2011 over all sampled sites in river red gum forests.
(DOC)
