# Supplementary figures and images for: Relating Demographic Characteristics of a Small Mammal to Remotely Sensed Forest-Stand Condition
Source: PLoS One. 2014 Mar 12;9(3):e91731. doi: 10.1371/journal.pone.0091731 (PMC3951454; doi:10.1371/journal.pone.0091731)

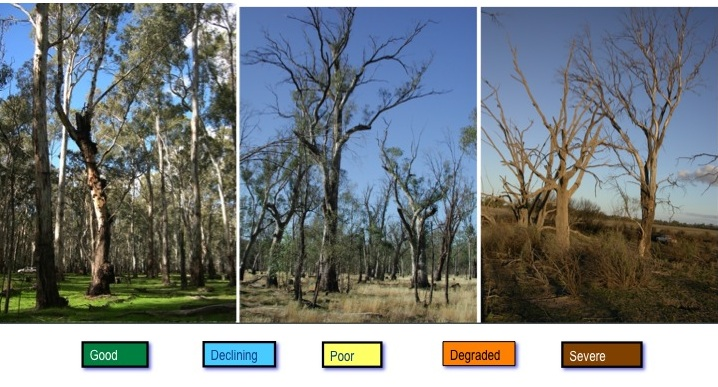

Supplement: Figure S1 — Examples of stand condition states: good, poor and severe. (TIFF) [file pone.0091731.s001.tiff]
