# Supplementary material for: Relating Demographic Characteristics of a Small Mammal to Remotely Sensed Forest-Stand Condition
Source: PLoS One. 2014 Mar 12;9(3):e91731. doi: 10.1371/journal.pone.0091731 (PMC3951454; doi:10.1371/journal.pone.0091731)
Supplement: File S1 — Contains the following files: Methods S1. Model selection with reversible jump MCMC. Stand-condition scores. Estimates of temporal trends in fractions of stand condition. References S1. Table S1 Study area and site information. Table S2 Mean trapping rates of the yellow-footed antechinus in 2004, 2005 and 2011 over all sampled sites in river red gum forests. (DOC) [file pone.0091731.s002.doc]

**File S1 Supporting Information**

**Methods S**

*Model selection with reversible jump MCMC*

We used Bayesian model selection, implemented with the reversible jump MCMC add-on to WinBUGS [1], to identify a subset of the *Q* = 7 candidate predictor variables that should be included in the best model, or, equivalently, to identify which of the linear coefficients *β* are non-zero. There is an unknown number *k* ≤ *Q* of potential predictors with non-zero coefficients. We assigned a binomial prior distribution to *k,* Binomial(0.5, *Q*), such that all combinations of predictor variables (all model structures) were equally probable a priori, implying a prior expectation of *Q*/2 variables in the model. With this prior, posterior probabilities are determined by marginal likelihoods that perforce favour simpler models [2].

Models were estimated with three Markov chain Monte Carlo (MCMC) chains run for 100000 iterations following burns-in of 50000 iterations. MCMC mixing and convergence were confirmed by inspection of chain histories, autocorrelation plots and Brooks-Gelman-Rubin statistics. We standardized (centred and divided by standard deviation) all predictor variables prior to model fitting to facilitate model convergence.

*Stand-condition scores*

Stand condition is estimated from the three variables: percentage live basal area, plant area index (PAI) and crown vigour, which are known to be reliable indicators of condition in *E. camaldulensis* stands [3]. Each variable is given equal weighting in calculating the stand-condition score, which has a maximum of ten points. PAI is the area of stems and leaves per unit ground area, unadjusted for clumping of canopy components. PAI was estimated from hemispherical photos using Winphot 5.00 [4] and then was standardized by the known local maxima. Crown extent is the percentage of potential crown, which is based on the observed branching structure that contains foliage. Crown extent was estimated using an interval scale (0%, 1-20%, 21-40%, 41-60%, 61-80%, 81-100%) from 30 representative trees. The diameters (at breast height, dbh) of all stems > 10 cm within the site were measured, from which basal areas were calculated. Percentage live basal area is the percentage of a stand’s basal area that is live trees, with trees considered alive if they had green leaves in their crown.

*Estimates of temporal trends in fractions of stand condition*

To track changes in stand condition across these floodplains, a temporal series (1990, 2003, 2006, 2009 and 2010) of condition maps for the region was compiled from previous work. Maps of stand condition across these floodplains developed from ground surveys in 2006 [5], 2009 [6] and 2010 [7], were used to hindcast stand condition in 1990 and 2003. To estimate stand condition in 1990 and 2003, stand-condition models were supplied with Landsat imagery from those years, assuming that the relationship between stand condition and reflectance data remains similar among years. For analysis, the maps were classified into 5 condition classes: good (*C* = 8.1–10.0), declined (*C* = 6.1–8.0), poor (*C* = 4.1–6.0), degraded (*C* = 2.1–4.0) and severe (*C* ≤ 2) condition using ArcGIS (ESRI, Redlands, California). Percentages of the total area in each condition class were determined from the number of pixels. The validation dataset (*N* = 42 stands) was used to determine the field accuracy of model predictions. The Bayesian models were used to estimate the 25% and 75% credible limits for the linear relationship between the predicted values of stand condition and those observed during the validation survey. The equations for the 25% and 75% credible limits were used to produce the ‘worst case’ and ‘best case’ predictions for stand condition classes across the study area.

**References S**

1. Lunn DJ, Best N, Whittaker JC (2005) Generic reversible jump MCMC using graphical models. pp. EPH-2005-01. London: Department of Epidemiology and Public Health, Imperial College.

2. Kass RE, Raftery AE (1995) Bayes Factors. Journal of the American Statistical Association 90: 773-795.

3. Cunningham SC, Read J, Baker PJ, Mac Nally R (2007) Quantitative assessment of stand condition and its relationship to physiological stress in stands of *Eucalyptus camaldulensis* (Myrtaceae). Australian Journal of Botany 55: 692–699.

4. ter Steege H (1996) WINPHOT 5.0: a programme to analyze vegetation indices, light and light quality from hemispherical photographs. Tropenbos Guyana Programme, Report 95-2. Tropenbos, Guyana.

5. Cunningham SC, Mac Nally R, Read J, Baker P, White M, et al. (2009) A robust technique for mapping vegetation across a major river system. Ecosystems 12: 207-219.

6. Cunningham SC, Mac Nally R, Griffioen P, White M (2009) Mapping the Condition of River Red Gum and Black Box Stands in The Living Murray Icon Sites. Stand Condition Report 2009 (with modelled results for 2003 and 2008). . Canberra: Murray-Darling Basin Authority.

7. Cunningham SC, Griffioen P, White M, Mac Nally R (2011) Mapping the Condition of River Red Gum (*Eucalyptus camaldulensis* Dehnh.) and Black Box (*Eucalyptus largiflorens* F.Muell.) Stands in The Living Murray Icon Sites. Stand Condition Report 2010. Canberra: Murray-Darling Basin Authority.

**Table S1** Study area and site information

| Forest Name | Sites centered on | Number of 0.25 ha sites | | | number of 500x500m within-forest areas | |
| --- | --- | --- | --- | --- | --- | --- |
|  |  | in 2004 | in 2005 | in 2011 | in 2004 | in 2005 |
| Ovens River | 36º 13' S, 146º 15' E | 13 | 13 |  | 1 | 1 |
| Barmah | 35º 53' S, 145º 01' E | 25 | 23 | 8 | 3 | 2 |
| Millewa | 35º 50' S, 145º 00' E | 22 | 29 |  | 3 | 3 |
| Koondrook | 35º 43' S, 144º 15' E | 27 | 27 |  | 3 | 3 |
| Gunbower Island | 35º 43' S, 144º 13' E | 101 | 105 | 16 | 12 | 13 |
| Guttrum | 35º 35' S, 144º 04' E | 23 | 22 |  | 1 | 1 |
| Campbells Island | 35º 34' S, 144º 04' E | 14 | 15 |  | 1 | 1 |
| Total |  | 225 | 234 | 24 | 24 | 25 |

**Table S2** Mean trapping rates of the yellow-footed antechinus in 2004, 2005 and 2011 over all sampled sites in river red gum forests.

| Year | Total | Males | Females | Second-year females | Second-year females with suckled teats |
| --- | --- | --- | --- | --- | --- |
|  | Mean ± SD | Mean ± SD | Mean ± SD | Mean ± SD | Mean ± SD |
| 2004 | 0.051 ± 0.013 | 0.032 ± 0.008 | 0.019 ± 0.006 | 0.003 ± 0.003 | 0.001 ± 0.001 |
| 2005 | 0.099 ± 0.025 | 0.067 ± 0.008 | 0.031 ± 0.010 | 0.006 ± 0.004 | 0.004 ± 0.003 |
| 2011 | 0.115 ± 0.054 | 0.077 ± 0.048 | 0.027 ± 0.015 | 0.004 ± 0.009 | 0.002 ± 0.005 |
